# Identification of Multiple Grain Shape-Related Loci in Rice Using Bulked Segregant Analysis With High-Throughput Sequencing

**DOI:** 10.3389/fpls.2020.00303

**Published:** 2020-04-03

**Authors:** Lian Wu, Yue Cui, Zhengjin Xu, Quan Xu

**Affiliations:** Rice Research Institute of Shenyang Agricultural University, Shenyang, China

**Keywords:** rice, grain shape, high-throughput sequence, BSA, *DEP1*

## Abstract

Grain shape (GS) is an important agronomic trait that can improve rice breeding for optimal appearance quality, and it varies highly between *indica* and *japonica* subspecies. In this study, we conducted a genome sequencing of a series of recombination inbred lines (RILs) derived from a cross between *japonica* variety Shennong265 (SN265) and *indica* variety R99, and we successfully detected seven loci associated with GS. Subsequent analysis showed that a major quantitative trait locus (QTL) corresponded to the *qSW5/GW5*. To identify a main-effect locus, we conducted bulked segregant analysis (BSA) in two F_2_ populations. A 0.8-Mb region on chromosome 9 was identified as the candidate region of GS. There are 101 predicted genes in this region, and eight single nucleotide polymorphisms/insertions and deletions (SNPs/INDELs) caused frameshift. We found that a 637-bp stretch in exon 5 of the *DENSE AND ERECT PANICLE 1* (*DEP1*) locus in SN265 was replaced by a 12-bp sequence. The two types of CRISPR/Cas9 gene-edited plants confirmed that *DEP1* affected GS, and both Gγ and Cys-rich domains participated in this regulatory mechanism. These findings improve our understanding of the underlying mechanism of GS in rice and provide an effective and rapid strategy for the identification of main-effect loci of target traits.

## Introduction

Rice, a staple food crop cultivated worldwide, feeds over 50% of the world’s population. GS is one of the most important agronomic traits that influences yield and quality. The *indica* and *japonica* rice varieties differ in term of GS, and this trait has undergone extensive selection during rice domestication and breeding. Recent studies have shown that GS is controlled by multiple signaling pathways, and advances in the functional genomics have facilitated the cloning of a series of loci that control grain size. For example, *qSW5/GW5* encodes a polyubiquitin-binding protein that is involved in the ubiquitin-proteasome pathway. This was shown to be a major loci of rice domestication and *indica/japonica* differentiation (Shomura et al., 2008; Weng et al., 2008). Phytohormones (i.e., cytokinins, brassinosteroids, and auxins) also participate in GS regulation. *GS5*, *GS2/GL2*, and *GW5* have been reported to be involved in brassinosteroid signaling (Li et al., 2011; Che et al., 2015; Hu et al., 2015). *GL3.1* regulates cyclin-T1-38 to influence GS, and *qGL3* encodes OsPPKL1phosphatase, which has been suggested to be involved in brassinosteroid signaling (Qi et al., 2012). *TGW6* encodes an IAA-glucose hydrolase activity protein, and *BG1* encodes a membrane localized protein; both may be specifically induced by auxin (Ishimaru et al., 2013; Liu et al., 2015a). In G-protein signaling pathways, GS3 is known to be a G protein γ subunit, which has been identified as a GS regulator (Fan et al., 2006; Takano-Kai et al., 2009; Mao et al., 2010). These studies have demonstrated that *GS3* is the most important determinant of grain length in breeding populations of cultivated rice, and the natural variants of *GS3* have contributed to the global improvement of rice yield and quality. Furthermore, transcriptional regulatory factors have also been shown to play important roles in the regulation of GS, including *GLW7*, *OsSPL16/GW8*, and *OsMKK4- OsMAPK5*. *GLW7* encodes the plant-specific transcription factor *OsSPL13* that positively regulates cell size (Si et al., 2016). *OsSPL16/GW8* directly binds to the *GW7/GL7* promoter and represses its expression via a SBP-domain transcription factor (Wang et al., 2012, 2015). The *OsMKK4- OsMAPK5* regulatory module also plays a key role in regulating GS by controlling cell proliferation of spikelet hulls (Duan et al., 2013; Liu et al., 2015b). Taken together, recent molecular studies have successfully identified numerous important genes that are associated with GS. However, their biological mechanisms and regulatory network remain largely unknown. Thus, identification and characterization of additional QTLs/genes that participate in the determination of GS is important not only to the elucidation of the molecular mechanisms underlying the regulation of grain traits but also to the generation of high yield and superior quality cultivars.

The present study conducted genome sequencing of a series of RILs that were derived from crossing the *indica* variety “R99” and the *japonica* variety “SN265” to identify the QTL/genes involved in the regulation of GS. Moreover, we conducted a BSA to identify the main-effect loci of GS. These findings improve our understanding of the underlying biological mechanisms of GS in rice and provide a rapid and cost-effective strategy for the identification of main-effect loci of target traits.

## Materials and Methods

### Plant Materials and Phenotype Determination

To identify the QTL/genes associated with GS, we generated an RIL population using the single-seed descendant method with at least 10 generations. The parent line was “R99” (*indica*) and “Shennong265” (*japonica*). A total of 151 RILs were constructed and used in the present study. The RILs were planted in three typical rice cultivated areas, namely, LN: Rice Research Institute of Shenyang Agricultural University (N41°, E123°), JS: the sub-base of China National Hybrid Rice R&D Center in Jiangsu Province (N32°, E120°), and SZ: the Agricultural Genomics Institute at Shenzhen (N22°, E114°) in the summer of 2016. The plots for each line were 5.4 m^2^ and included 120 plants with planting densities (hill per m^2^) of 22.2 (30 cm × 15 cm intervals). Plots were arranged in a randomized block design with three replications. The cultivation method and field management were as described in our previous report (Li et al., 2018). Twenty plants from the middle plot were harvested for each line at 45 days after heading. The plant materials for BSA were planted at the Rice Research Institute of Shenyang Agricultural University (SY; N41°, E123°) in 2018. To determine the GS of the experimental materials, we harvested the paddy rice and air-dried these at room temperature for at least 3 months before testing. Fully filled grains were used for measuring grain length, width, thickness, and weight. Ten randomly selected grains from each plant were lined up lengthwise along a Vernier caliper to measure grain length, and they were then arranged by breadth to measure grain width.

### DNA Extraction and High-Throughput Sequencing

The young leaves of each line were sampled 15 days after transplanting. The cetyl trimethylammonium bromide (CTAB) method was used to obtain the high-quality genomic DNA. We constructed the sequencing libraries on the Illumina HiSeq2500 according the manufacturer’s instructions. The sequence data was aligned with the Nipponbare genome, as referenced^1^ by SOAP2. The HighMap software was used to combine the co-segregating SNP/InDel into bin for linkage map. The QTL analysis was conducted using R/qtl (version: 1.44-9) software via a composite interval mapping (CIM) model. The details of the linkage map construction and QTL analysis were described in our previous study (Li et al., 2018).

### The Details of BSA

We developed two DNA bulks by selecting extreme short-grain plants and extreme long-grain plants in the F_2_ populations. We then prepared the libraries for the DNA bulks following the Illumina TruSeq DNA sample Preparation v2 Guide. The MiSeq Reagent Kit v2 (500 cycles) of Illumina MiSeq platform (Illumina Inc., San Diego, CA, United States) was used to sequence the DNA libraries. We then aligned the short reads of the DNA bulks along with the parent lines to a Nipponbare reference genome (IRGSP 1.0) by the BWA software (Li and Durbin, 2009). To call SNPs, the reads of the two bulks were separately aligned to SN265 and R99 consensus sequence reads using SAM tools (Li and Durbin, 2009). We removed duplicate reads using the Picard tool according to the clean reads mapping results using the genome reference^2^. We performed the base recalibration and local realignment to guarantee the accuracy of SNP detection using GATK software (Mckenna et al., 2010). The SNPs between the reference genome and samples were detected by the GATK software^3^. We calculated the SNP-index and Euclidean distance (ED) to identify the candidate regions that are associated with GS (Hill et al., 2013). The ED algorithm’s calculation formula is as follows

E⁢D=(T⁢m⁢u⁢t-T⁢w⁢t)2(A⁢m⁢u⁢t-A⁢w⁢t)2+(C⁢m⁢u⁢t-C⁢w⁢t)2+(G⁢m⁢u⁢t-G⁢w⁢t)2+2,

where *Amut*, *Cmut*, *Gmut*, and *Tmut* are the bases A, C, G, and T’s frequency in the long-grain-type bulks, respectively. *Awt*, *Cwt*, *Gwt*, and *Twt* are the frequency of bases A, C, G, and T in the short-grain-type bulks, respectively. To eliminate the background noise, the ED value was powered, and the associated value was set as ED^5^. Then, we fit the data using a Loess curve with a polynomial exponent of 1 and a span parameter determined by minimizing the Aikaike Information Certerion (AICc). Peak regions are defined as regions where the Loess fitted values are greater than three standard deviations above the genome-wide median (Hill et al., 2013). SNP-index association analysis was used to calculate differences in genotype frequency between the two bulks (Rym et al., 2013; Takagi et al., 2013).

### CRISPR/Cas9 Gene Editing

We performed vector construction to conduct CRISPR/Cas9 gene editing as described by Li et al. (2017). We selected the targeting sequence, including the PAM sequence (23 bp) in the first and fifth exons of the *DEP1* gene. By performing a BLAST search against the rice genome, we confirmed the specificity of the targeting sequence^4^ (Hsu et al., 2013). We performed rice transformation as described elsewhere (Nishimura et al., 2006). The genomic DNA of the transformants was sequenced and analyzed using the Degenerate Sequence Decoding method (Ma et al., 2015).

## Results

### Phenotypic Characterization of Grain Shape in SN265 and R99

**FIGURE 1:**
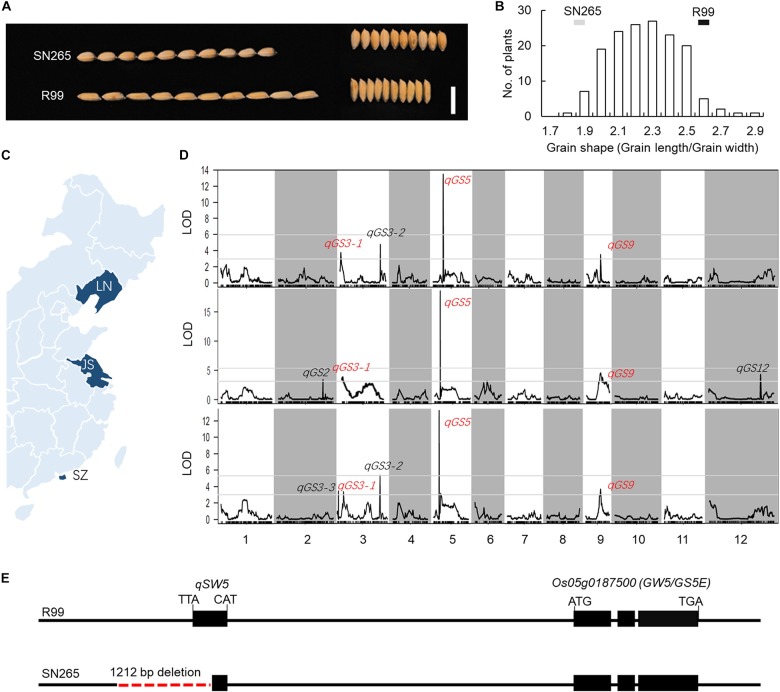
QTL analysis and candidate gene. **(A)** GS of SN265 and R99. Bar = 1 cm. **(B)** The distribution of GS in RILs. **(C)** The plant area of RILs **(D)** QTL analysis of GS. **(E)** The difference in the *GW5/GS5E* locus between SN265 and R99.

In the present study, the *indica* variety “R99” and the *japonica* variety “SN265” were used to identify GS-related loci. A notable difference in GS was observed between SN265 and R99 (Figure 1A). The grain length of SN265 was 5.18 ± 0.03 mm, whereas that of R99 was 6.13 ± 0.03 mm. The grain widths of SN265 and R99 were 2.75 ± 0.01 mm and 2.33 ± 0.03 mm, respectively. The GS (grain length/grain width) of SN265 was 1.88 ± 0.02, whereas that of R99 was 2.6 ± 0.02. SN265 and R99 were then crossed and inbred for over 10 generations to develop the RIL population for QTL analysis of GS. The GS of RILs showed a typical normal distribution. Some lines exhibited transgressive segregation, which suggests that GS was controlled by multiple loci in this cross combination (Figure 1B).

### QTL Analysis of GS-Related Loci Using RILs

To detect the loci that are related to GS in the RILs, we planted the RILs in three areas and conducted QTL analysis via high-throughput sequencing (Figure 1C). Our previous study constructed a high-density bin map using the Highmap software (Li et al., 2018). The molecular linkage map, which consisted of 3,569 bins, was used to conduct the QTL analysis of GS. A total of seven loci were detected with a LOD >2.5 and were located on chromosomes 2, 3, 5, 6, 9, and 12, and some of these QTLs could be detected in multiple areas (Figure 1D; Supplementary Table S1). The LOD of one major QTL *qGS5* on chromosome 5 ranged from 12.44 to 17.32; and this QTL explained 31.38–42.13% of the observed variations in three areas. Within this region, we found the key GS regulator *qSW5/GW5*, which has been shown to be the major GS loci for rice domestication and *indica/japonica* differentiation (Shomura et al., 2008; Weng et al., 2008). We then compared the *qSW5/GW5* sequence in SN265 and R99. The results show that there was a 1,212-bp deletion in SN265 when compared with R99 (Figure 1E). These results indicate that *qSW5/GW5* might be the target QTL/gene of the major QTL *qGS5* detected in SN265/R99 RIL population in this study.

### BSA of Main-Effect Loci of GS

**FIGURE 2:**
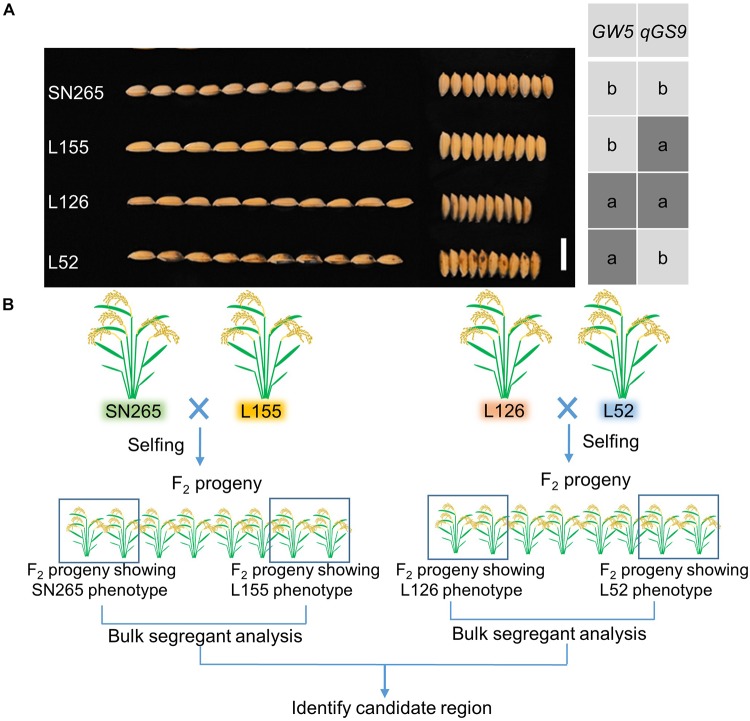
The population construction for BSA. **(A)** The GS of SN265 and L155. Bar = 1 cm. **(B)** Simplified scheme for the application of BSA.

**FIGURE 3:**
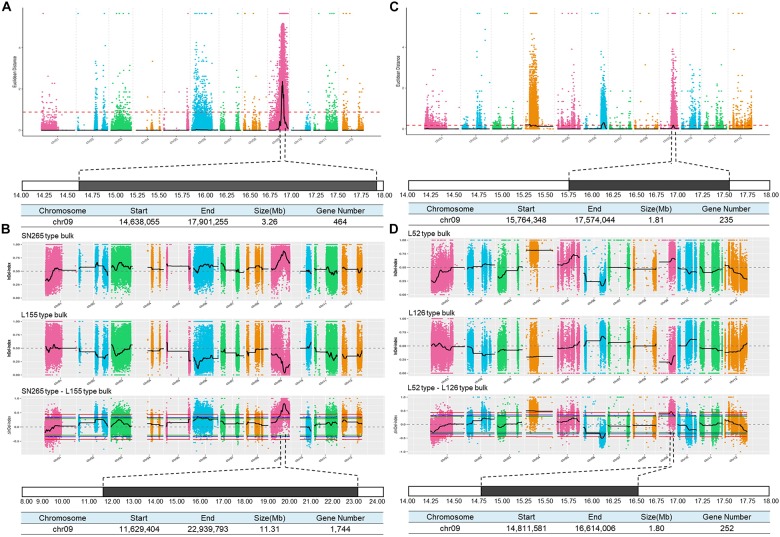
BSA analysis of GS. **(A)** Identification of the hot-region for GS via ED association analysis of the cross between SN265 and L155. **(B)** Identification of the candidate gene for GS via the SNP-index method of the cross between SN265 and L155. **(C)** Identification of the hot-region for GS via ED association analysis of the cross between L126 and L52. **(D)** Identification of the candidate gene for GS via the SNP-index method of the cross between L126 and L52.

**FIGURE 4:**
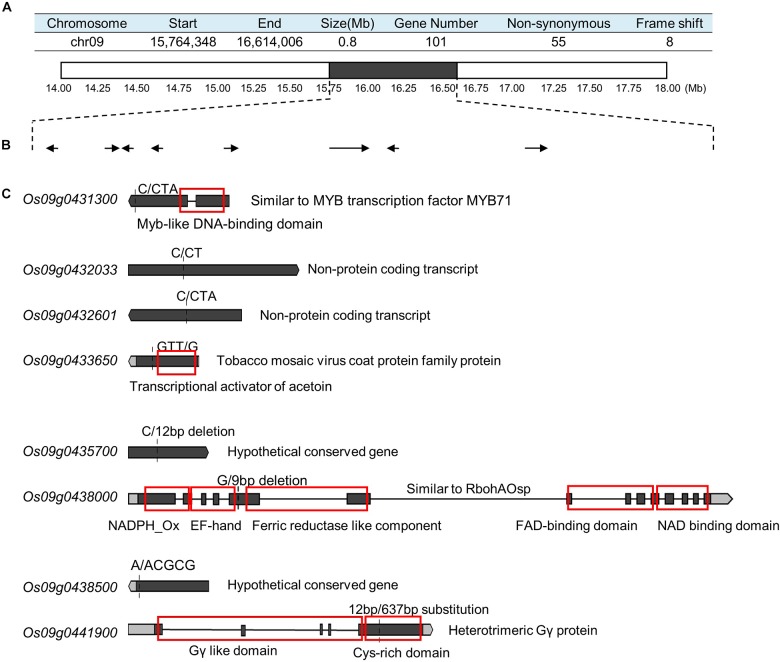
Identification of candidate gene. **(A)** The candidate region identified by ED and SNP-index methods using two pairs of BSA populations. **(B)** The genes in which the SNP/InDel result in a frame shift. **(C)** The detail of sequence analysis of the eight genes. The red boxes indicate the functional domains.

To detect the main-effect QTLs involved in GS regulation, we subsequently focused on the QTL located on chromosome 9 (*qGS9*) that could explain 11.32–12.63% of the observed variations (LOD = 3.64–4.40) and could be detected in all three areas (Figure 1D). We conducted the parental line selection based on genotypic data at *qSW5/GW5* and *qGS9*. Line 155 (L155) shared the same type of *qSW5/GW5* but a different allele of qGS9 compared to that of SN265. Moreover, the grains of L155 were significantly slimmer than SN265. Additionally, we found that L126 and L52 shared a R99-type allele at the *qSW5/GW5* locus but differed in *qGS9*. L126 has shorter and wider grains than L152 (Figure 2A). Then, we crossed the L155 to SN265, and crossed L126 to L52 to develop two pairs of bulks for BSA analysis. We first sampled 30 plants that exhibited short grains as SN265 and long grains as L155 as bulks for high-throughput sequencing, respectively (Figure 2B). The Illumina high-throughput sequencing generated 70.91 Gb of data, covering 97.47% of whole genome. There was an average of 47.08-fold for each sample. There were 922,924 SNPs between SN265 and L155, and 22,119 of these were non-synonymous. A total of 116,993 SNPs were detected between the two bulks, and 1,860 of these were non-synonymous. We also detected 194,851 and 30,797 small INDELs between parental lines and between the two bulks, respectively. To improve the accuracy of BSA analysis, we used two approaches, the ED and SNP-index methods, to identify the candidate regions related to GS (Figure 3). The association threshold of the ED method was indicated by pink broken lines in Figure 3A, and only a region 3.26 Mb in length was significantly correlated with GS. Then, we calculated the SNP index of the two bulks for each SNP and computed an average SNP-index at a 1-Mb interval using a 10-kb sliding window; an 11.11-Mb region was identified that was significantly correlated with GS (Figure 3B). Thus, we obtained a 3.26-Mb candidate region by combining the result of ED and SNP-index methods using the F_2_ population from the cross between SN265 and L155. We subsequently sampled 30 plants having GS similar to L52 and 30 plants showing similar GS as L126 as two bulks for BSA. The sequencing generated 70.91-Gb of data, covering 97.18% of whole genome. There was an average of 47.08-fold genomic coverage for each sample. There were 784,722 SNPs between L126 and L52, and 19,902 of them were non-synonymous. Using the ED method, we detected a 1.81-Mb region from 15,764,348 to 17,574,044 on chromosome 9 that significantly correlated to GS (Figure 3C). By SNP-index methods, we identified a 1.80-Mb region from 14,811,581 to 16,614,006 on chromosome 9 related to GS (Figure 3D). Taken together, we obtained a 0.8-Mb candidate region using the ED and SNP-index method for the two pairs of the BSA population (Figure 4A). The candidate region consisted of 101 genes with 56 non-synonymous SNPs (Supplementary Table S2). Our previous study performed the *de novo* assembly of two high-quality genomes of SN265 and R99, which are the parental lines in the present study (Li et al., 2018). Thus, we can thoroughly compare the sequences of the 0.8-MB chromosome region between SN265 and R99. Among genes containing 56 non-synonymous SNPs, eight genes that exhibited frame shift mutations were chosen as candidate genes that might be more probably associated with GS (Figure 4B). Among the 56 non-synonymous SNPs, we subsequently found eight genes with frame shift mutations, and these eight genes were probably associated with GS (Figure 4B). *Os09g0432033*, *Os09g0432601*, *Os09g0435700*, and *Os09g0438500* were non-protein coding transcript or hypothetically conserved genes. *Os09g0431300* contained a Myb-like DNA-binding domain at the N-terminus, and the C/CTA difference between SN265 and R99 occurred at the C-terminus of the *Os09g0431300*. Similarly, the GTT/G difference in *Os09g0433650* avoided the transcriptional activator of acetoin domain in *Os09g0433650.* A 9-bp deletion was detected in *Os09g0438000* between the EF-hand domain and Ferric reductase-like component domain, but the deletion did not influence these two functional domains. Interestingly, there is a 637-bp stretch in the exon 5 of *Os09g0441900* in the R99 genome that was replaced by a 12-bp sequence in SN265 (Figure 4C). This gene has been reported as a pleiotropic locus for panicle architecture and grain number, named as *DENSE AND ERECT PANICLE 1* (*DEP1*) (Huang et al., 2009; Wang et al., 2009; Zhou et al., 2009). The substitution caused an elimination of Cys-rich domain at the C-terminus of *DEP1. DEP1* might thus be the candidate gene for this main-effect QTL affecting GS.

### Confirmation Using CRISPR/Cas9 Gene Editing Technology

**FIGURE 5:**
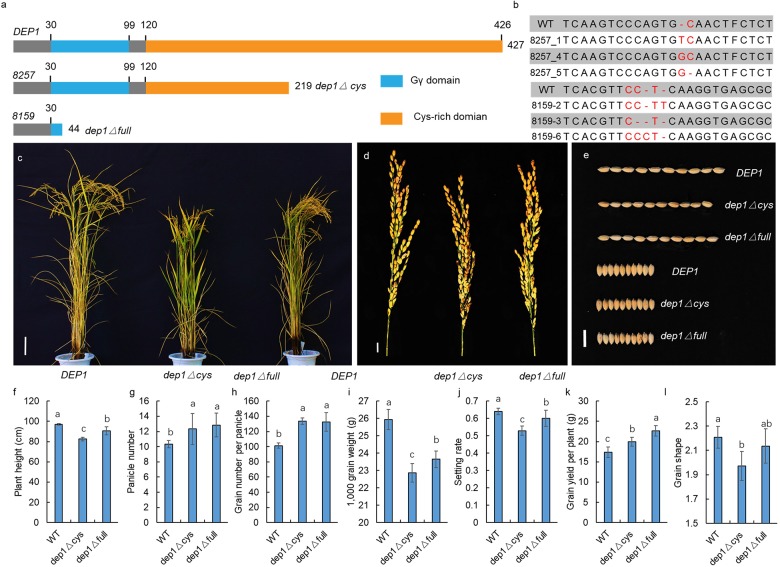
Confirmation by CRISPR/Cas9 gene editing technology. **(a)** The protein structure of *DEP1*, *dep1*△ *cys*, and *dep1*△ *full*. **(b)** Sequence mutation of the CRISPR/Cas9 gene edited plant. **(c)** The plant architecture of *DEP1*, *dep1*△ *cys*, and *dep1*△ *full*. **(d)** The panicle of *DEP1*, *dep1*△ *cys*, and *dep1*△ *full*. **(e)** The GS of *DEP1*, *dep1*△ *cys*, and *dep1*△ *full*. **(f–l)** The plant height, panicle number, grain number per panicle, 1,000 grain weight, setting rate, grain yield per plant and GS of *DEP1*, *dep1*△ *cys*, and *dep1*△ *full* plants. Data are means ± SE (*n* = 20), different letters denote significant differences (*P* < 0.05, Duncan multiple-range test).

To confirm the function of *DEP1* in GS regulation, we generated a series of *dep1* mutations using the CRISPR/Cas9 gene editing technology under the genetic background of Sasanishiki (a famous *japonica* variety) as wild-type control (WT). The *DEP1* has a Gγ at its N-terminus and a Cys-rich domain at its C-terminus (Figure 5a). As a previous study showed that the truncated *dep1* allele that lost the cys-rich domain was a gain-of-function mutant, we generated two types of *dep1* mutants. As shown in Figure 4A, the *dep1*△*cys* allele loses the Cys-rich region but maintains the Gγ domain, whereas the *dep1*△*full* allele loses both the Cys-rich and Gγ domains. At least three independent transgenic lines were obtained (Figure 5b). The results show that the *dep1*△*cys* plants exhibited a semi-dwarf and erect panicle architecture, and panicle length and grain length decreased relative to the WT (Figures 5c–e). The *dep1*△*full* plants showed a slight decrease in plant height, panicle length, and grain length. No significant differences in grain width were observed among WT, *dep1*△*cys*, and *dep1*△*full.* These results indicate that the *DEP1* locus indeed participates in the regulation of GS, and both the Gγ and the Cys-rich domains played essential roles in GS regulation.

## Discussion

Recent molecular studies have identified a number of major QTL/genes that control GS. However, further efforts to identify main-effect QTL/genes are necessary. Our study used genome sequencing and a BSA strategy to successfully identify seven QTLs that were related to GS. Among the QTLs, one major locus *qGS5* was co-located with *GW5/GS5E*, which has been reported to be related to rice grain width and grain weight. Weng et al. (2008) and Shomura et al. (2008) fine mapped *GW5* to a 2,263-bp and 21-kb genomic region, respectively. Subsequent studies demonstrated that a gene encoding a calmodulin-binding protein, which was located approximately 5 kb downstream of the 1,212-bp deletion, corresponded to *qSW5/GW5* (Duan et al., 2017; Liu et al., 2017). Our study confirmed *qSW5/GW5* as a major locus that explained 42.13% of the observed variation in the JS experimental site as the highest. The G protein has been shown to possess the ability to regulate GS and rice yield. The *GS3*, a Gγ subunit protein-encoding gene, is a major QTL for GS, and it functions as a negative regulator of GS and organ size (Fan et al., 2006; Mao et al., 2010). Another Gγ subunit protein-encoding gene, *RGG2*, has been reported to participate in the regulation of GS and rice yield (Miao et al., 2019). Sun et al. (2018) used multiple mutation combinations of the G protein encoding gene to propose a model to explain how these Gγ proteins work in GS regulation. Taken together, the G-protein pathway plays an important role in GS regulation. Our study also detected QTLs in chromosome 9 that were associated with GS and explained 11.35% of the observed variations, and the additive effects ranged from 0.06–0.07 among different experimental sites. The candidate gene was identified as *DEP1* using the BSA strategy. The *DEP1* locus was first reported as a panicle architecture regulator by three research teams (Huang et al., 2009; Wang et al., 2009; Zhou et al., 2009). A subsequent study showed that *DEP1* is also involved in nitrogen utilization (Sun et al., 2014). A “self-inhibition” model was constructed at the C-terminal domain that inhibited the N-terminal domain in non-canonical Gγ proteins in *DEP1* (Mao et al., 2010; Botella, 2012). Recent molecular studies employing CRISPR/Cas9 gene editing have been conducted to verify the function of truncated *DEP1* (Li et al., 2016, 2018, 2019; Shen et al., 2017; Wang et al., 2017; Sun et al., 2018). The plants with a Gγ domain but no cys-rich domains exhibited an erect panicle architecture and an increase in the grain number per panicle. The plants that had lost both the Gγ and cys-rich domains, however, showed reductions in grain number per panicle and setting rate. Additionally, the truncated *dep1* allele also exhibited a negative effect in grain number per panicle under the genetic background of Wuyunjing 8 and Non-gken 57 (Zhou et al., 2009; Yi et al., 2011). These results suggested that the truncated *dep1* alleles generated an opposite phenotypic change on grain number per panicle under a different genetic background. The present study showed that both the *dep1*△*cys* and *dep1*△*full* plants exhibited an increase in grain number per panicle compared to the WT (Figure 5). Li et al. (2019) showed that three lines harboring different lengths of truncation at the C-terminal of *DEP1* by CRISPR/Cas9 gene editing technology exhibited similar phenotypic changes of the GS. Taken together, we hypothesize that both the *dep1*△*cys* and *dep1*△*full* could increase grain number per panicle, generate a semi-dwarf phenotype, and reduce panicle length and grain length. Since the 1980s, a number of high-yielding *japonica* rice strains with dense and erect panicles have been released as commercial varieties. In China, these *japonica* ideotypes, such as SN265 and Jiahua, 1 have dominated the *japonica* rice acreage, and almost all of these varieties harbored the truncated allele of *DEP1*, which is similar to *dep1*△*cys* (Zhao et al., 2016). Interestingly, we found that the *dep1*△*full* transgenic plants that lost both Gγ and Cys-rich domains exhibited an enhanced phenotype compared to the WT and *dep1*△*cys* plants (Figures 5f–l). These findings suggest a potential method to develop important germplasm with novel allele of *DEP1* locus and suggest a novel strategy for the flexible utilization of *DEP1* in rice breeding.

## Data Availability Statement

The datasets supporting the conclusions of this study are included within the report.

## Author Contributions

QX and ZX designed this study and contributed to the original concept of the project. LW and YC performed most of the experiments. QX wrote the manuscript. All of the authors have read and approved the final manuscript.

## Conflict of Interest

The authors declare that the research was conducted in the absence of any commercial or financial relationships that could be construed as a potential conflict of interest.

## Footnotes

1 https://rapdb.dna.affrc.go.jp/download/irgsp1.html

2 http://sourceforge.net/projects/picard/

3 https://www.broadinstitute.org/gatk/guide/best-practices.php

4 http://blast.ncbi.nlm.nih.gov/Blast.cgi
